# FOLFOX Chemotherapy Ameliorates CD8 T Lymphocyte Exhaustion and Enhances Checkpoint Blockade Efficacy in Colorectal Cancer

**DOI:** 10.3389/fonc.2020.00586

**Published:** 2020-04-23

**Authors:** Yue Guan, Sean G. Kraus, Michael J. Quaney, Mark A. Daniels, Jonathan B. Mitchem, Emma Teixeiro

**Affiliations:** ^1^Department of Molecular Microbiology and Immunology, School of Medicine, University of Missouri, Columbia, MO, United States; ^2^Department of Surgery, School of Medicine, University of Missouri, Columbia, MO, United States; ^3^Informatics Institute, University of Missouri, Columbia, MO, United States; ^4^Research Service, Harry S. Truman Memorial Veterans' Hospital, Columbia, MO, United States

**Keywords:** chemotherapy, colorectal cancer, CTL, TIL, exhaustion, immune checkpoint blockade

## Abstract

**Background:** Colorectal cancer (CRC) is the third most common malignancy worldwide. The presence of CD8 tumor-infiltrating T lymphocytes (TILs) is associated with improved prognosis and therapeutic response in CRC patients. FOLFOX chemotherapy is a standard first-line treatment for patients with CRC. Yet, the effect of FOLFOX on TILs is poorly understood. Specifically, it is unclear whether FOLFOX therapy impacts the phenotype and functionality of tumor antigen specific TILs. Immune checkpoint blockade (ICB) has significantly improved clinical outcome of cancer treatment but has shown limited efficacy in CRC patients. Recently, ICB efficiency has been linked to reinvigoration of T cells with a non-terminally dysfunctional phenotype. Here, we investigate the effect of FOLFOX on CD8 T cell tumor accumulation, phenotype and function and tested the combination of FOLFOX and ICB to improve tumor regression.

**Methods:** A mouse model of CRC expressing a human tumor antigen was used to study the effect of FOLFOX on tumor growth and TILs phenotype and function. Tetramers were used to identify and monitor phenotype and function of tumor specific TILs. The phenotype and function of TILs were compared between FOLFOX and control treatment through flow cytometry, *in vivo* depletion and *ex vivo* stimulation. Furthermore, the anti-tumor effect of the single drug or combined therapy with anti-PD1 were also assessed.

**Results:** We show that FOLFOX treatment effectively controlled tumor burden and this was dependent on CD8 T cells. FOLFOX enabled TILs to remain in a functional differentiation state characterized by lower levels of inhibitory receptors PD-1 and TIM-3 and a CD38^lo^CD101^lo^TIM-3^−^TCF-1^hi^ phenotype. Consistent with this, TILs from FOLFOX treated tumors exhibited higher effector function. Importantly, while anti-PD-1 treatment alone had no significant effect on tumor burden, FOLFOX and PD-1 checkpoint blockade combination showed significant tumor control.

**Conclusions:** FOLFOX treatment impacts the phenotype and function of TILs making them more responsive to checkpoint blockade. This study highlights the importance of combining chemotherapy and ICB to optimize treatment efficacy in patients with colorectal cancer.

## Introduction

Colorectal cancer (CRC) is the second leading cause of cancer death in the United States today, with approximately 50,000 deaths per year (1). While recent advances in immune based therapy have significantly improved cancer related outcomes in patients with other malignancies, these results have not yet extended to the majority of patients with CRC (2). This is somewhat surprising given increasing evidence that suggests the immune response is important for survival, recurrence, and therapeutic efficacy in CRC patients (3–5). Hence, understanding how to effectively utilize immune based therapy in CRC is critical for future treatment development.

Cytotoxic CD8 T lymphocytes (CTLs) are the primary mediators of anti-tumor immunity. In tumors, however, CD8 tumor-infiltrating lymphocytes (TILs) are often unable to function effectively due to tumor induced immune suppression or acquired unresponsiveness (6–8). One specific mechanism of CTL dysfunction in the tumor microenvironment is exhaustion. Exhaustion is supported by prolonged exposure to TCR signaling by cognate antigen (7, 9). Chronic exposure to antigen in the tumor microenvironment leads to high expression of inhibitory receptors, such as PD-1, TIM-3, CTLA-4, and LAG-3, as well as a gradual loss of the ability to produce effector cytokines (IFN-γ, TNF-α, and IL-2) and proliferate (7, 9). Interestingly, it has been shown that CTLs become dysfunctional very early during tumorigenesis through an antigen driven dynamic differentiation program (10). In this program, CTLs acquire epigenetic and phenotypic traits that are associated with plastic vs. fixed dysfunctional stages (11). Exhausted CTLs include “progenitor exhausted” cells that retain polyfunctionality, persist long-term and provide better control of the tumor than “terminal exhausted T cells”. Phenotypically, “progenitor,” and “terminal” exhausted T cells are different and can be distinguished by the levels of expression of the inhibitory protein TIM-3 and the transcription factor TCF-1 (12).

Relief of exhaustion in tumor infiltrating lymphocytes is currently under intensive study as a therapeutic method due to significant advancements in the care of patients with melanoma, non-small cell lung cancer, and others using PD-1 and CTLA-4 checkpoint blockade (13). However, the majority of patients does not respond nor achieve durable responses to this therapy, including most CRC patients (14–18). This may be related to the plastic vs. terminal dysfunctional stage of patients' TILs. In agreement with this, melanoma patients with a higher percentage of “progenitor” exhausted cells (less exhausted and more plastic) have been recently shown to experience longer responses to checkpoint blockade therapy (12). Thus, finding therapeutic strategies that expand or maintain TILs in this progenitor exhausted reprogrammable stage could help to increase the number of patients that benefit from checkpoint therapy.

Accumulating evidence indicates chemotherapeutic agents have immune-modulatory effects (19, 20). The current primary first-line therapy for the treatment of patients with CRC is combination therapy with 5-fluorouracil (5-FU), leucovorin, and Oxaliplatin (Oxa), or FOLFOX (21). Both of the individual arms of this regimen, 5-FU and Oxa, have been shown to improve anti-tumor immunity (22–24). To develop effective combination strategies that capitalize on the benefits of both chemotherapy and immune based therapy, we sought to investigate the immune effects of this chemotherapeutic regimen in the context of CRC.

In this study, we show that FOLFOX chemotherapy increases both the quantity and quality of tumor antigen specific CTLs. Furthermore, and contrary to the common belief that chemotherapeutic agents only act through direct killing of tumor cells, our data shows that FOLFOX distinctively modulates CD8 anti-tumor immunity to reduce tumor burden. We show that FOLFOX chemotherapy modulates the level of exhaustion of TILs. FOLFOX treatment decreased the frequency of terminally exhausted TILs and increased the frequency of TILs with the progenitor exhausted phenotype that has been linked to better responses to immune checkpoint blockade (ICB) in melanoma (12). Confirming this, we show that PD-1 blockade therapy works more effectively in CRC models when combined with FOLFOX chemotherapy. The findings in this study support the idea that FOLFOX enables the accumulation of tumor specific CD8 T cells that are associated with favorable clinical response and prevents them from developing an irreversible dysfunctional differentiation program. These data have significant implications for the rational design of synergistic approaches involving conventional and current immunotherapies as well as the development of clinical trials in CRC patients.

## Materials and Methods

### Mice

C57BL/6J mice and BALB/c mice were purchased from the Jackson laboratory. Females and males were used in the studies, with no significant differences between genders. T lymphocyte deficient mice (B6.CD3d^−/−^ MHC class II^−/−^ MHC class I^−/−^) (25) were bred and housed in the animal facility of the University of Missouri [generous gift of Dr. Adam Schrum, University of Missouri (26)]. All animal experiments were conducted according to procedures reviewed and approved by the Institutional Animal Care and Use Committees in the University of Missouri.

### Cell Lines

The C57BL/6 syngeneic colorectal cancer cell line MC38 expressing antigen CEA2 (27) (MC38-CEA2) was purchased in 2017 from Kerafast, Inc. and cultured in DMEM supplemented with 10% FBS, 2 mM glutamine, 0.1 mM non-essential amino acids and 0.1 mM sodium pyruvate and 50 μg/ml gentamycin sulfate and 300 μg/ml G418. To study the impact of chemotherapy on tumor cells *in vivo*, MC38-CEA2 cells were engineered for stable expression of green fluorescent protein (GFP) (described below). The colorectal carcinoma cell line CT26 was purchased in 2017 from ATCC and cultured in RPMI complete media (RPMI 1640 supplemented with 10% FBS, 2 mM glutamine, 0.1 mM non-essential amino acids and 0.1 mM sodium pyruvate). Cells were used within 5 passages for all the experiments. Cell lines were authenticated by manufacturers tests.

### Generation of MC38-CEA2-GFP Cell Line

MC38-CEA2-GFP cell line was generated through retroviral transduction using the retroviral construct pMX expressing green fluorescent protein (GFP, pMX-EGFP) as described in Knudson et al. (28). Retrovirally transduced tumor cells were sorted twice with MoFlo XDP cell sorter (Beckman Coulter) to achieve 100% expression of GFP. MC38-CEA2-GFP cell line has same doubling time and expresses similar levels of surface CEA as the parental MC38-CEA2. The GFP is stably expressed and when injected in mice, the time of tumor onset and growth rate *in vivo* was identical to the parental cells.

### Murine Tumor Models

For MC38-CEA2 tumor model, 5 × 10^5^ MC38-CEA2 or MC38-CEA2-GFP cells suspended in 50 μl PBS were injected subcutaneously into the flank of C57BL/6 mice or T cell deficient hosts (CD3d^−/−^ MHC class II^−/−^ MHC class I^−/−^), and tumor growth was monitored every 2 or 3 days. Tumor dimensions were measured with digital caliper and size was calculated as length × width. Mice were sacrificed at indicated time points for analysis. CT26 colon cancer cells (5 × 10^5^ cells in 50 μl PBS) were also injected subcutaneously into BALB/c mice and tumor growth was measured using caliper measurements as described above. Each experimental group contains 4–10 animals.

### *In vivo* CD8 T Lymphocyte Depletion

For depletion of CD8 T lymphocytes, tumor-bearing mice were injected with anti-CD8 (clone 2.43, BioXcell) or isotype control Ab (clone LFT-2, BioXcell) via i.p. injection on the day before tumor implantation and continued every 4 days after for 3 weeks. The first antibody injection contained 500 μg of antibody diluted in 100 μl PBS and subsequent injections contained 250 μg of antibody in the same volume. The efficacy of CD8 T cell depletion was verified by flow cytometry through tail bleed at least once a week to ensure the depletion was maintained (≈ 100%).

### *In vivo* Tumor Treatment

After tumor establishment (>15 mm^2^, day 10 post inoculation), tumor-bearing mice were treated with PBS or chemotherapy (FOLFOX) once a week for 3 weeks. The dose given was: 5-Fu (100 mg/kg) and Oxa (2.5 mg/kg). Leucovorin was omitted as this adds marginal effectiveness to 5-FU but can significantly increase toxicity in murine subjects. The drugs were diluted in 100 μl PBS and given i.p. These doses are adopted from previous studies (23, 29) and are within the range (mg/Kg) used in the clinic for humans. For anti-PD-1 treatment, when tumors were established (~40 mm^2^, day 14 post inoculation), animals were assigned to treatment groups. Anti-PD-1 treatment was initiated with FOLFOX on the same day. Mice were treated with i.p. injection of anti-PD-1 (RMP1-14, BioXcell) or control antibody (clone 2A3, BioXcell) at 200 μg per mouse for a total of 6 doses every 3 days. FOLFOX was given once a week as described above. Tumor dimensions were measured and monitored every 2–3 days until endpoints.

### Isolation of Cells From Tumor and Murine Tissue

MC38-CEA2 and MC38-CEA2-GFP tumor tissues were harvested, weighed and minced into small pieces. Samples were suspended in RPMI 1640 containing collagenase (1 mg/mL; Sigma-Aldrich) and DNase I (100 μg/mL; Sigma-Aldrich) and transferred to MACS tubes and further disrupted on a gentle MACS dissociator (program mTumor-01). Samples were incubated for 30 min at 37°C, then filtered through a 70 μm nylon cell strainer (VWR) and pelleted by centrifugation. Pelleted tumor digest was suspended in 35% percoll solution (Sigma-Aldrich) and centrifuged at 1,600 rpm for 7 min to remove extra fatty tissue. Spleen and lymph node tissues were meshed and filtered through a 40 μM cell strainer and red blood cells lysed. Cells were counted and suspended in PBS supplemented with 0.5% BSA for flow cytometry staining.

### Flow Cytometry

Cell concentration was determined using 123 count eBeads (eBioscience). Cells were first incubated with Fc-block prior to antibody staining. The following antibodies were used from Biolegend: CD8 (53-6.7), CD4 (RM4-5), CD25 (PC61), CD44 (IM7), CD62L (MEL-14), PD1 (29F.1A12), TIM-3 (RMT3-23), CD38 (90), TNF-α (MP6-XT22), F4/80 (BM8), CD103 (2E7), CD45 (104), CD11c (N418), MHCII (M6/114.15.2), Ly6G (HK1.4), CD11b (M1/70); from BD Bioscience: Ki67, Ki67 isotype (RTK4530), and IFN-γ (XMG1.2); from Cell Signaling: TCF1 (C63D9) and Anti-rabbit 2nd; from Invitrogen: CD101 (Invitrogen, Moushi101) and FoxP3 (FJK-16s). Tumor antigen-specific T cells were stained with H2-Db/CEA_526−533_ EAQNTTYL tetramer (NIH Tetramer Core Facility). Intracellular staining was performed using Cytofix/Cytoperm kit (BD). For tumor cells, the following antibodies were used: CEA (B1.1, BD Bioscience), H2-Db (KH95, Biolegend), Calreticulin (polyclonal, ThermoFisher), PD-L1 (10F.9G2, Biolegend), and CD45 (104, Biolegend). Cells were run on LSRFortessa (BD) flow cytometer and data were analyzed in FlowJo X. The analysis for tumor cells was on GFP+CD45– population.

### *Ex vivo* CD8 T Cell Effector Function

For intracellular cytokine staining, freshly isolated cells from tumor or spleen were plated at up to 3 million cells/100 μl in 96-well plate and were stimulated with PMA (50 ng/ml, Sigma) and Ionomycin (500 ng/ml, Sigma) in the presence of Golgiplug (BD Bioscience) for 5 h followed by surface and intracellular staining as described in Knudson et al. (30). Stained cells were run on LSRFortessa (BD) flow cytometer and data were analyzed in FlowJo X as above.

### Statistical Analysis

**Figure 1:**
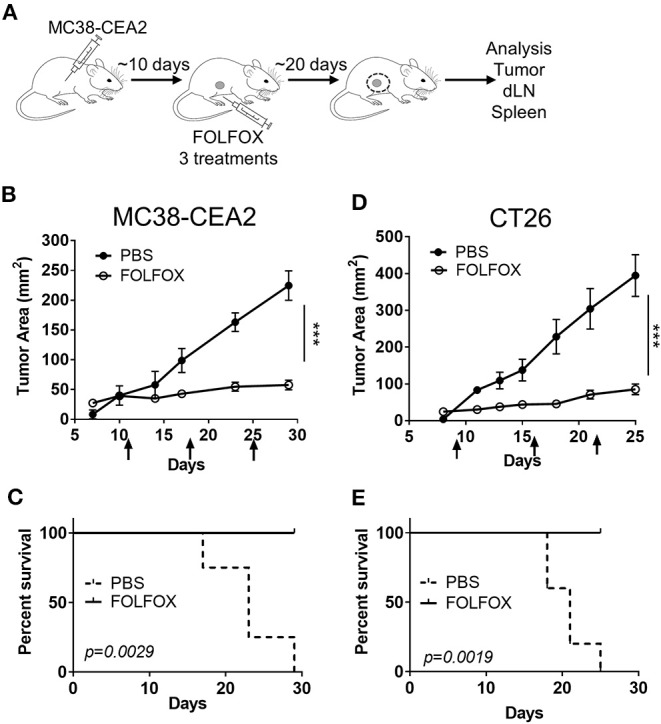
FOLFOX treatment slows colorectal tumor progression and prolongs survival. **(A)** Experimental scheme and treatment plan. MC38-CEA2 or CT26 cells were implanted subcutaneously in naïve C57BL/6 mice or BALB/c mice, respectively. After tumor was established, mice were treated with FOLFOX or PBS once weekly via intraperitoneal injection and tumor size was monitored. Arrows indicate dates of treatment. At the end of treatment, tumor tissue, tumor-draining lymph node (dLN) and spleen are collected and analyzed. **(B–E)** Graph shows means and SEM of tumor area for *n* ≥ 4 mice per group. Data is representative of *n* ≥ 3 independent experiments with each group containing 4–10 mice for MC38-CEA2 **(B)** and CT26 **(D)** CRC tumor models. Survival of mice treated with FOLFOX in MC38-CEA2 tumor model **(C)** and CT26 tumor model **(E)**. ***p < 0.001.

Statistical comparisons include one-way ANOVA followed by Tukey's comparison tests, unpaired two-tailed Student's *t*-tests and two-way ANOVA for Figures 1, 4, 6. Survival curve comparison was performed using the Log-rank (Mantel-Cox) test. *P*-values of 0.05 or less were considered statistically significant.

## Results

### FOLFOX Treatment Delays Colorectal Tumor Progression and Prolongs Survival

To determine the effect of FOLFOX on the anti-tumor immune response, we used a murine CRC cell line expressing the model antigen Carcinoembryonic Antigen (MC38-CEA2) (31) to track tumor antigen specific CD8 T cell immunity. Tumor cells were implanted subcutaneously in naïve C57BL/6 mice. After tumors were established (>15 mm^2^), mice were treated with intraperitoneal (IP) FOLFOX once every week for a total of 3 weeks and tumor growth was monitored for 30 days (Figure 1A). FOLFOX treatment significantly reduced tumor growth and extended survival (Figures 1B,C). However, the individual components of FOLFOX, 5-Fluorouracil (5-FU) and Oxaliplatin (Oxa), failed to effectively curb tumor growth (Figure S1). We obtained similar results in the CT26 colon carcinoma model (Figures 1D,E). Together these indicate that FOLFOX effectively controls tumor growth and prolongs survival in murine CRC models similar to what is seen in human patients. Since FOLFOX therapy is a current first-line therapy in CRC patients (21) and significantly outperformed single agent therapy (Figure S1), we then focused on the effect of FOLFOX on anti-tumor immunity.

### FOLFOX Treatment Increases the Presence of Tumor Antigen Specific CD8 T Cells in the Tumor

**Figure 2:**
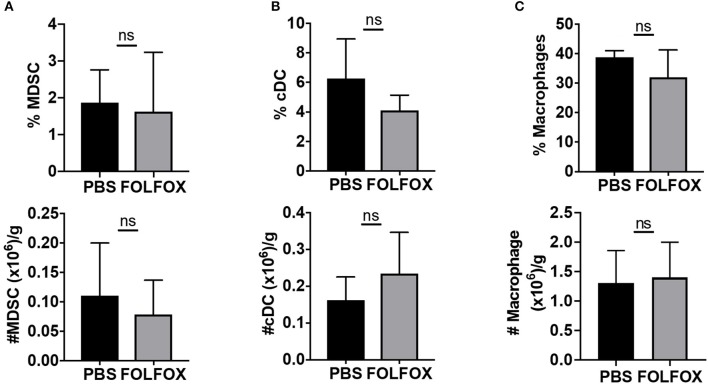
FOLFOX treatment has no effect on the presence of tumor infiltrated myeloid populations. Phenotypic identification of tumor-infiltrating myeloid cell subsets harvested 4 days after the third treatment in experiments performed as in Figure 1 by flow cytometry. Myeloid cells subsets, **(A)** MDSC (CD11b+Gr1+), **(B)** cDC (CD11b+ CD11c+ F4/80–), and **(C)** Macrophages (CD11b+ CD11c– F4/80+) are presented as the frequency and absolute number per gram of tumor tissue.

We next determined whether FOLFOX chemotherapy altered the composition of tumor-infiltrating cells. Both 5-FU and Oxa have been shown to distinctly affect immune cells in the tumor microenvironment (22, 23). Using a previously described gating strategy (32) (Figure S2A), we first assessed FOLFOX-dependent changes in the tumor associated myeloid cell compartment. Myeloid cells (CD45+CD8-CD11b+) were subdivided into myeloid derived suppressor cells, MDSC (Gr1+), macrophages (F4/80+CD11c+) and conventional DCs (CD11c+F4/80–). 5-FU has previously been shown to selectively decrease MDSC (22). However, when comparing FOLFOX and PBS treatments, we found no significant difference in the frequency or number of tumor-associated MDSC or other myeloid subsets described above (Figures 2A–C).

**Figure 3:**
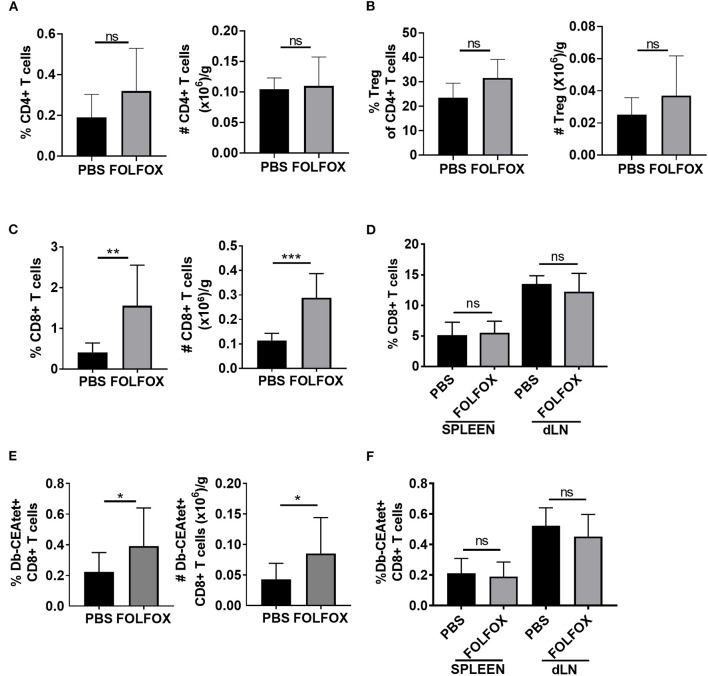
FOLFOX treatment is associated with an increase in tumor antigen-specific CTLs. Phenotypic identification of tumor-infiltrating lymphocytes subsets harvested 4 days after the third treatment in experiments performed as in Figure 1 by flow cytometry. **(A)** CD4 T cells, **(B)** regulatory T cells or Treg (CD4+CD25+FoxP3+), and **(C)** CD8 T cells are presented as the frequency and absolute number per gram of tumor tissue. **(D)** Spleen and dLN in MC38-CEA2 tumor-bearing mice were analyzed for CD8 T cell frequency. **(E,F)** Tumor antigen-specific CTLs were gated on CD8+CD44+Db-CEAtet+. Graphs show the frequency (over live gate) and number of CEA specific CD8 T cells in tumor **(E)** and the frequency of CEA specific CD8 T cells in spleen and dLN **(F)** in untreated (PBS) and FOLFOX treated mice. Representative data of n≥3 experiments with each group containing 4–6 mice. *p < 0.05, **p < 0.01, ***p < 0.001.

Then, we examined the effect of FOLFOX treatment on the T cell compartment. CD4 T lymphocyte frequencies and numbers were not altered upon FOLFOX treatment. Additionally, we found no difference in regulatory T cells (Treg) (Figures 3A,B and Figure S2B). By contrast, when compared to untreated controls, FOLFOX treatment led to an increase in the frequency and number of tumor infiltrating CD8 T cells in both the MC38-CEA2 colorectal model and the CT26 colorectal model (33) (Figure 3C and Figure S3). This effect was limited to the tumor microenvironment, as CD8 T cell frequencies were not significantly different in the spleen or tumor draining lymph nodes (dLN) (Figure 3D).

Since clearance of tumors is related to the presence of tumor antigen specific CD8 T cells in the tumor, we also determined the effect of FOLFOX treatment on their frequency in CEA expressing tumors. The frequencies and numbers of CD8 T cells specific for the tumor antigen CEA (CD44^hi^CD8^+^Db-CEA tetramer^+^) were greater in FOLFOX-treated mice than in controls (Figure 3E and Figure S2B). Again, we found no differences in spleen and dLNs upon FOLFOX treatment (Figure 3F). Collectively, these results show that FOLFOX treatment increases the presence of tumor antigen specific CD8 T cells in the tumor.

In addition, we observed that FOLFOX treatment increased surface expression of calreticulin (CRT) on tumor cells, a key determinant in immunogenic cell death-driven cancer immunity (34). Furthermore, the level of MHC class I expression (MHCI, Db) was also augmented on tumor cells (Figures S4A,B). Together these data are consistent with the idea that exposure of CRT leads to engulfment of cancer material by dendritic cells, tumor antigen presentation and anti- tumor CTLs specific responses (35). These results also suggest that FOLFOX, similar to other chemotherapy regimens (23), may promote immunogenic cell death and contribute to the expression of tumor antigen and the activation of innate immunity necessary for the infiltration and the activation of tumor specific CD8 T cells in the tumor.

### FOLFOX Requires CD8 T Cells for Optimal Tumor Control

**Figure 4:**
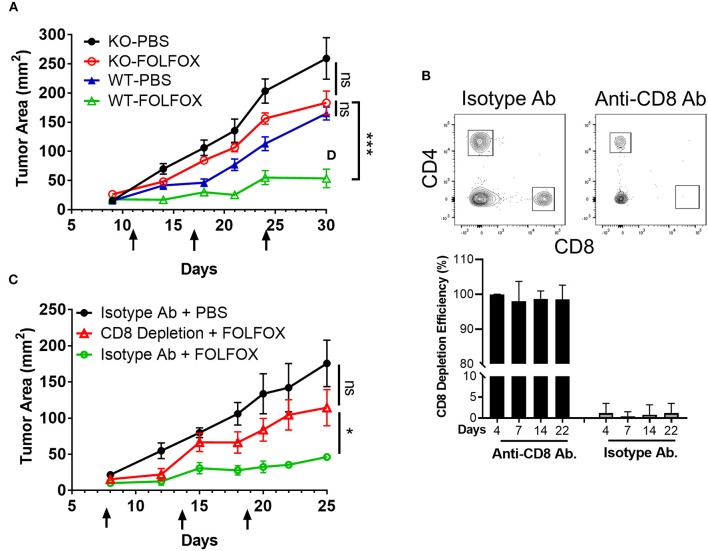
FOLFOX-mediated tumor control depends on the presence of CD8 T lymphocytes. **(A)** MC38-CEA2 tumor cells were implanted in T lymphocyte deficient hosts (CD3d^−/−^MHC class II^−/−^, MHC class I^−/−^) or WT mice. Tumor bearing mice were treated with PBS or FOLFOX and tumor size was monitored and plotted over three treatment cycles. Arrow indicates days at which mice received treatment. Graph shows means and SEM of tumor area for *n* = 3–5 mice per group. Data is representative of 3 independent experiments. **(B)** Mouse CD8 T lymphocytes were depleted using anti-CD8 depleting antibody. Anti-CD8 or isotype antibodies were given the day before tumor implantation and continued for every 4 days during 3 weeks. Dot plots (day 14) and graph show anti-CD8 T cell depletion efficiency based on detection of CD8 T cells in blood (%) by flow cytometry at the days indicated. **(C)** Tumor growth was monitored and plotted over three treatment cycles. Graph shows means and SEM of tumor area for *n* = 3–5 mice per group. Data is representative of three independent experiments. *p < 0.05, ***p < 0.001.

We then evaluated to what extent T cells were required for FOLFOX efficacy. To test this, we first implanted MC38-CEA2 tumor cells in T cell deficient hosts. T cell deficient mice bearing MC38-CEA2 tumors were then treated with FOLFOX or PBS as outlined in Figure 1. In T cell deficient mice, FOLFOX marginally controlled tumor growth with no significant difference between treated and untreated mice (Figure 4A). Furthermore, in experiments where approximately 100% of CD8 T cells where depleted from mice harboring tumors (Figure 4B), FOLFOX failed to significantly suppress tumor growth (Figure 4C). Strikingly, these data show that CD8 T cell mediated anti-tumor immunity is critical for the therapeutic effect of FOLFOX in CRC.

### FOLFOX Treatment Promotes a CD8 T Cell Phenotype That Is Associated With Less Exhaustion

**Figure 5:**
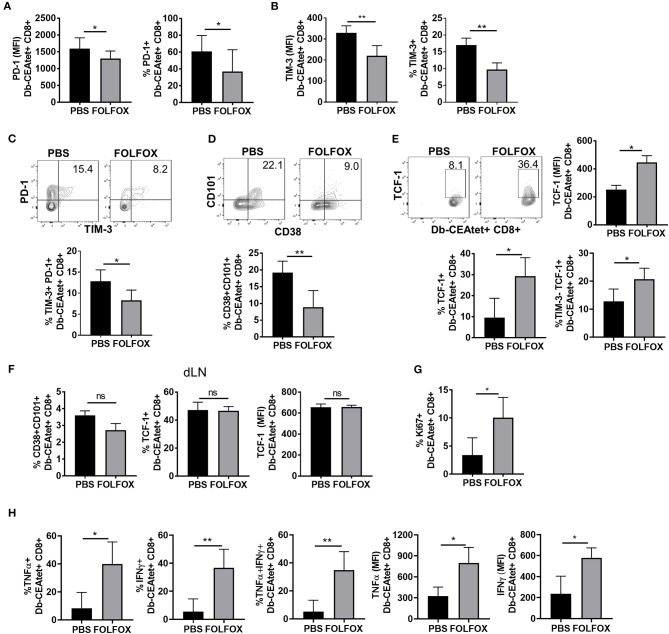
Upon FOLFOX treatment, TILS are phenotypically less exhausted and more functional. Tumor antigen specific CD8 T cells from PBS or FOLFOX treated animals harvested 4 days after the third treatment were analyzed for indicated phenotype. Tumor antigen specific CD8 T lymphocytes were gated on CD8+CD44hiDb-CEAtet+ population. Graphs show frequency of tumor antigen specific CD8 T cells that are positive for the expression of PD-1 **(A)** or TIM-3 **(B)** or both **(C)**. Levels of PD-1 **(A)** and TIM-3 **(B)** expressed by tumor antigen specific CD8 T cells were assessed by MFI in flow cytometry analysis. **(C)** Representative flow cytometric analysis of PD-1 and TIM-3 expression of CEA specific TILs where numbers indicate %. **(D–F)** Representative flow cytometric analysis of CD38, CD101, and TCF-1 expression of CEA specific TILs where numbers indicate %. Graphs show frequencies of terminally exhausted (CD101+CD38+CD8+ Db-CEAtet+) **(D)** or TCF-1 positive CD8 T cells (TCF-1+Db-CEAtet+CD8+) or “progenitor exhausted” phenotype (TIM-3-TCF1+Db-CEAtet+CD8+) or levels of TCF-1 (MFI) **(E)** in tumor antigen specific CD8 T cells over control levels. **(F)** Exhaustion phenotype in tumor antigen specific CD8 T cells in draining lymph nodes. **(G)** Tumor antigen specific CD8 T cells were analyzed for expression of Ki67. Cells were first gated on CD8+ CD44+ Db-CEA tetramer+ Ki67+ based on its isotype control staining. **(H)** Tumor cells were re-stimulated with PMA and ionomycin for 5 h in the presence of Golgiplug for *ex vivo* T cell function analysis. Graphs show frequency of CEA antigen specific CD8 T cells that are positive for the expression of the cytokine indicated and levels of the cytokines. Representative data of ≥3 with each group containing 4–6 mice. *p < 0.05, **p < 0.01.

Tumor antigen specific CD8 T cells that are constantly exposed to tumor antigen gradually transition into a dysfunctional stage that renders terminally exhausted T cells, unable to respond to the tumor. Indeed, relief of CD8 T cell exhaustion is a crucial area of study in ICB based cancer therapy. Transition to T cell exhaustion is characterized by gradual loss of effector function, proliferation, and permanent high expression of inhibitory receptors (such as PD-1 and TIM-3). Given the increased presence of tumor antigen specific CD8 T cells in FOLFOX-treated tumors (Figure 3), we evaluated their level of exhaustion. First, we determined the expression of inhibitory receptors PD-1 and TIM-3 (36, 37), as numerous studies in mice and humans have correlated their level of expression with the extent of T cell exhaustion (38, 39). We found that the expression of both PD-1 and TIM-3 was significantly lower on tumor antigen specific CD8 T cells in FOLFOX-treated tumors (Figures 5A,B). The frequency of PD-1+ and TIM-3+ tumor specific CD8 T cells was also significantly lower in FOLFOX-treated tumors (Figure 5C). However, this was not the case in dLN and Spleen (Figure S5).

Exhausted or dysfunctional T cells represent a distinct immune lineage with broad heterogeneity. This heterogeneity has been attributed to the progressive and dynamic development of T cell exhaustion that occurs as TILs continue to receive antigenic signals in the tumor. More importantly, not all subsets of exhausted T cells are the same, with some phenotypes associated with better tumor control and lower functional exhaustion (“progenitor exhausted”) while others are associated with terminal exhaustion and no response to ICB (“terminal exhausted”) (10, 12, 39, 40). Thus, we asked whether FOLFOX had any impact in the exhaustion phenotype of TILs in our CRC model (40). Tumor specific CD8 T cells with plastic and fixed exhaustion programs can be distinguished phenotypically depending on the expression of CD101 and CD38 (high in terminally exhausted CD8 T cells) and the transcription factor TCF-1 (low in terminally exhausted CD8 T cells) (11). Interestingly, we found that the frequency of CD101+CD38+ tumor specific CD8 T cells was significantly lower in cells isolated from the tumors of FOLFOX-treated mice. Furthermore, tumor antigen specific TILs also expressed higher levels of the transcription factor TCF-1 (Figures 5D,E), indicating that upon FOLFOX treatment TILs are less exhausted. Remarkably, the frequency of TILs with the recently described “progenitor exhausted “phenotype (12) (TIM-3- TCF-1+) that is associated with better antitumor responses was also higher in FOLFOX-treated tumors (Figure 5E). Tumor antigen specific CD8 T cells in dLNs, however, showed a very low frequency of CD8 T cells associated with a terminally exhausted phenotype (CD38+CD101+ TCF1lo). Moreover, there were no differences in the phenotype regardless of FOLFOX treatment (Figure 5F). Additionally, we observed an increase in the frequency of progenitor exhausted cells in the tumor (low in TIM-3+ or high in TCF1+) that occurred between the second and third treatment of FOLFOX (Figure S6A). Together these data indicate that FOLFOX treatment favors the generation and/or maintenance of TILs expressing a profile of inhibitory receptors and a surface and transcriptional phenotype associated with low levels of T cell exhaustion.

### Increased Effector Function of Tumor Infiltrating Lymphocytes Upon FOLFOX Treatment

Terminally dysfunctional or exhausted CD8 T cells are characterized for their inability to respond to stimulation. Since, FOLFOX treatment rendered a higher frequency of TILs with a less exhausted phenotype, we tested the function of these cells. First, we determined the proliferative response of tumor antigen specific TILs by measuring the expression of Ki67, a cell-cycle marker expressed by cycling or recently divided cells (41). We found an increase in Ki67 positive tumor antigen-specific CD8 T cells isolated from FOLFOX-treated tumors, indicating that CD8 T cells in FOLFOX-treated tumors are more proliferative in the tumor (Figure 5G). We next assessed the ability of tumor antigen specific CD8 T cells to induce the expression of effector cytokines as a readout of their functional response to stimulation. We observed a 5–7- fold increase in the frequency of tumor antigen specific CD8 T cells expressing IFNγ and TNFα in FOLFOX-treated tumors compared to controls. More importantly, tumor antigen-specific CD8 T cells expressed higher levels of both cytokines (Figure 5H) and this was readily seen after the 2nd FOLFOX treatment (Figure S6B). On the contrary, total and tumor antigen specific CD8 T lymphocytes isolated from the spleen were not significantly different (Figure S7). Thus, consistent with the less exhausted phenotype, TILs exhibited a greater functionality upon FOLFOX treatment.

### FOLFOX Increases the Efficacy of PD-1 Checkpoint Blockade to Improve Tumor Control

**Figure 6:**
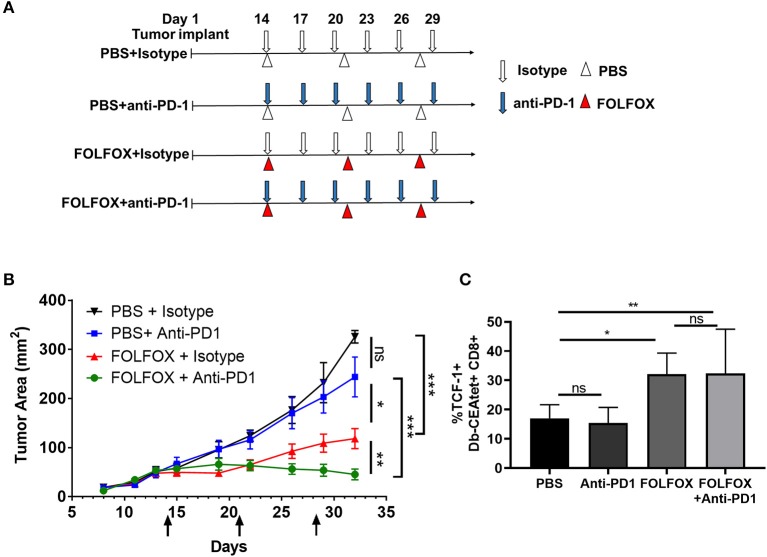
FOLFOX improves immune checkpoint blockade treatment on colon cancer. **(A)** Scheme of treatment. MC38-CEA2 cells were implanted subcutaneously in naïve C57BL/6 mice. After tumor was well-established (40–70 mm^2^ in size), FOLFOX and anti-PD-1 treatment were started on the same day. Mice were injected (i.p.) with anti-PD-1 antibody (200 μg/mouse) every 3 days for 3 weeks. FOLFOX or PBS were given once weekly via i.p injection for 3 weeks. **(B)** Graph shows means and SEM of tumor area for *n* = 3–5 mice per group. **(C)** The frequencies of TCF-1 positive CD8 T cells (TCF-1+Db-CEAtet+CD8+) in the indicated treatment. Data is the result of combining two independent experiments. *p < 0.05, **p < 0.01, ***p < 0.001.

Our data so far indicated that FOLFOX treatment favor the infiltration of functional TILs with a TIM-3- TCF1+ “progenitor exhausted” phenotype. Recently, TIM-3– TCF-1+ tumor infiltrating CD8 lymphocytes have been shown to be the most efficient target of checkpoint blockade therapy in melanoma (12). To test this in our CRC model, we compared the effect of the combination of FOLFOX and checkpoint blockade vs. each one of the independent treatments on tumor burden over time. Consistent with the reported low efficiency of anti-PD-1 on colorectal cancer patients (2), MC38-CEA2 mouse tumor did not respond to anti-PD-1 treatment. Strikingly, the combination of FOLFOX and anti-PD-1 significantly improved tumor control compared to any of the treatments alone (Figures 6A,B). This was in consonance with the change in the exhaustion phenotype of TILs that we have observed upon FOLFOX treatment (Figure 5). Indeed, while anti-PD-1 treatment did not increase the frequency of TILs with a progenitor exhausted phenotype (TCF1+), FOLFOX and combination FOLFOX and anti-PD-1 treatment did (Figure 6C). Thus, these data suggest that FOLFOX treatment can positively modulate the lack of response to anti-PD1 monotherapy.

## Discussion

In this study, we report that FOLFOX chemotherapy significantly increases tumor-associated CD8 T cell number and function by impeding the acquisition of a terminal exhaustion or dysfunctional program. More importantly, our data suggest that FOLFOX-dependent support of a low exhaustion TIL phenotype can improve the efficacy of PD-1 checkpoint blockade therapy in CRC. To our knowledge, this is the first report showing that chemotherapy changes the level of TIL exhaustion in CRC. Historically, the anti-tumor effect of chemotherapy has been attributed to a direct influence on rapidly dividing tumor cells. Accumulating evidence, however, strongly suggests that the anti-tumor effects of chemotherapy also involve host immunity (19, 20). Indeed, our data indicate that not only does FOLFOX induce an increase in tumor CD8 T cell infiltration, but most importantly, tumor control mediated by FOLFOX is abrogated in the absence of CD8 T cells. Studies in lung cancer models have also shown that other specific chemotherapy regimens lead to increased CD8 T cell infiltration due to immunogenic cell death (ICD), which is known to increase inflammation and tumor antigen presentation in the tumor (23).

The data presented here indicates that the effects of FOLFOX on tumor antigen specific CD8 T cells go beyond the well-studied role of ICD and point to a profound effect of chemotherapy on the differentiation of CD8 T cells in the tumor. CD8 T cell dysfunction in tumors has recently been demonstrated as a distinct differentiation state driven by persistent antigen exposure. The terminal dysfunction of tumor CD8 T cells is regulated by epigenetic and transcriptional programs marked by up regulation of CD38 and CD101 and decreased expression of TCF-1 (10, 11). In our model, we found that, consistent with increased effector function, tumor-associated CD8 T cells in FOLFOX-treated mice expressed lower levels of inhibitory receptors (PD-1 and TIM-3). More importantly, those cells also had lower levels of CD38 and CD101, and increased TCF-1, the signature associated with reprogrammable CD8 T cell exhaustion. These results suggest an effect of FOLFOX on tumor infiltrating lymphocytes that has not been revealed before and could be exploited therapeutically to either relieve T cell exhaustion or to maintain tumor-infiltrating lymphocytes in a plastic state amenable to immune checkpoint blockade. In this line tumor antigen-specific CD8 T cells with high levels of the transcription factor TCF-1 respond better to anti-PD-1 checkpoint blockade (11, 12). Our finding that FOLFOX and PD-1 blockade combination therapy provided significantly better tumor control in CRC than either therapy alone illustrates the potential of chemotherapy regimens to improve the response to checkpoint blockade therapies in cancer. This suggests a potentially critical function of FOLFOX that renders immunologically unresponsive tumors susceptible to immune directed approaches. This is highly important for the development of future clinical trials. Similarly, a recent study by Dosset et al. showed that a single co-administration of FOLFOX and anti-PD-1 was sufficient to completely eradicate tumors in the murine CT26 CRC model while FOLFOX treatment alone was insufficient (42). Interestingly, the authors of this study reported that FOLFOX triggers tumor adaptive immune resistance based on increased expression of PD-1 in TILs and PDL-1 in tumor cells, suggesting that the blockade of PD-1 breaks this resistance. In our studies we also found that PD-L1 was upregulated on tumor cells with FOLFOX treatment. However, our data shows that FOLFOX, in the presence of CD8 T cells, reduces tumor burden. Furthermore, FOLFOX treatment increased the number of tumor antigen specific T cells in the tumor that are polyfunctional and retain a non-terminally exhausted phenotype (11, 43).

A critical component for the development of T cell exhaustion is persistent exposure to antigen (10). However, other processes in the tumor microenvironment can also lead to exhaustion. For example, the effect of chemotherapy limiting tumor cell growth and consumption of available nutrients in the tumor microenvironment may help to deter the development of T cell exhaustion. Nutrient depletion and hypoxia have both been shown to drive CD8 T cell dysfunction in the tumor microenvironment (44, 45). In fact, CD8 T cells stimulated in the presence of PD-1 ligation alters metabolism to enhance fatty acid oxidation and repress glycolysis to prevent effector differentiation (44). Additionally, PD-L1 blockade in another model restored CTL glycolytic capacity and effector function (45). Thus, it is possible that FOLFOX treatment limits tumor cell metabolism while inducing ICD, thereby increasing the presence of CTLs and preventing their exhaustion. Future studies are needed to fully understand how the multiple effects of FOLFOX and other chemotherapy coordinate to modulate anti-tumor immunity and compare them with other regimens.

Our study shows that FOLFOX-based chemotherapy regulates the exhaustion stage of tumor-infiltrating CD8 T cells. This has important implications for the combination of FOLFOX and immune checkpoint blockade as there are current trials utilizing FOLFOX with combination immune checkpoint inhibitor therapy in metastatic CRC (46). Since FOLFOX therapy maintains tumor specific CD8 T cells in a non-dysfunctional/terminally exhausted state, this may provide an additive or synergistic effect to current immune checkpoint blockade in human patients, which we demonstrate in our tumor model. Due to an ability to improve the amount of CTLs to the tumor and prevent the development of tumor associated CD8 T cell dysfunction, FOLFOX may be highly effective in combination with CAR-T cell and adoptive cell transfer (ACT) therapy. This has critical importance for the treatment of patients with CRC, as immune based therapies, including immune checkpoint blockade, have demonstrated limited efficacy in the majority of these patients (2). Only CRC patients with mismatch repair deficiency or microsatellite instability-high (MSI-H) tumors have been shown to respond to PD-1 therapy. These tumors are characterized by high numbers of tumor infiltrating lymphocytes, believed to be driven by increased neoantigen load and enhance TIL responses (2, 18). Future studies should consider the effect of MSI-H status on the response to FOLFOX.

In summary, we present in this study significant evidence that FOLFOX chemotherapy requires CD8 T cells for optimal therapeutic efficacy and maintains CD8 tumor infiltrated lymphocytes in a differentiation stage that is still functional and more amenable to ICB. This suggests a potentially important role for FOLFOX in the immunologic activation of CRC tumors, rendering these tumors susceptible to immune-based therapy. These data may have a significant impact for the future development of clinical trials combining specific chemotherapy regimens and immune based therapy for the treatment of patients with CRC.

## Data Availability Statement

The datasets generated for this study are available on request to the corresponding author.

## Ethics Statement

The animal study was reviewed and approved by Institutional Animal Care and Use Committees in the University of Missouri.

## Author Contributions

JM and ET: study concept and design. YG, SK, and ET: acquisition, analysis, or interpretation of data. YG, JM, and ET: drafting of the manuscript. MD, YG, and ET: Critical revision of the manuscript for important intellectual content. YG: statistical analysis. JM and ET: obtained funding. YG, SK, MQ, and MD: administrative, technical, or material support. JM and ET: study supervision.

## Conflict of Interest

The authors declare that the research was conducted in the absence of any commercial or financial relationships that could be construed as a potential conflict of interest.
