# Supplementary material for: FOLFOX Chemotherapy Ameliorates CD8 T Lymphocyte Exhaustion and Enhances Checkpoint Blockade Efficacy in Colorectal Cancer
Source: Front Oncol. 2020 Apr 23;10:586. doi: 10.3389/fonc.2020.00586 (PMC7190812; doi:10.3389/fonc.2020.00586)
Supplement: Supplementary file 1 [file Data_Sheet_1.PDF]

Figure S1

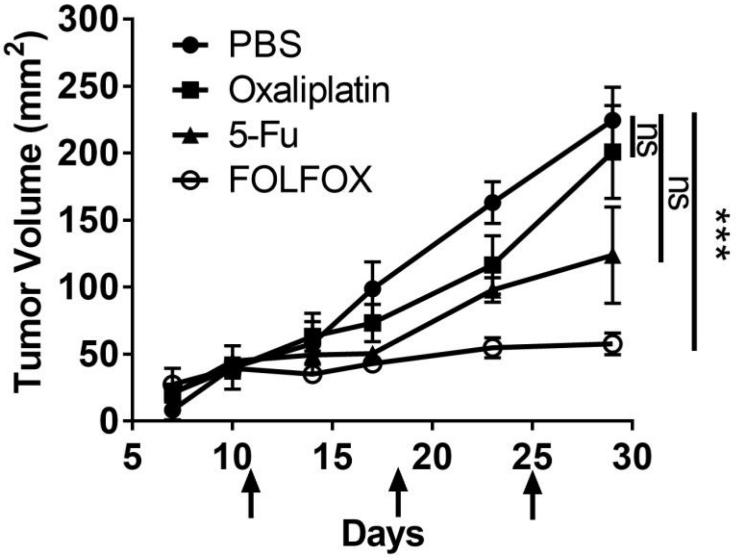

Figure S1. **FOLFOX treatment significantly slows colorectal tumor progression and prolongs survival.** Mean tumor burden of MC38-CEA2 implants. MC38-CEA2 was implanted subcutaneously in naïve C57BL/6 mice. After tumor was established, mice were treated with indicated chemotherapy twice weekly via intraperitoneal injection and tumor size was monitored. Arrows indicate dates of treatment. Representative data of multiple experiments with each group containing 4-10 mice. ***p<0.001.

Figure S2

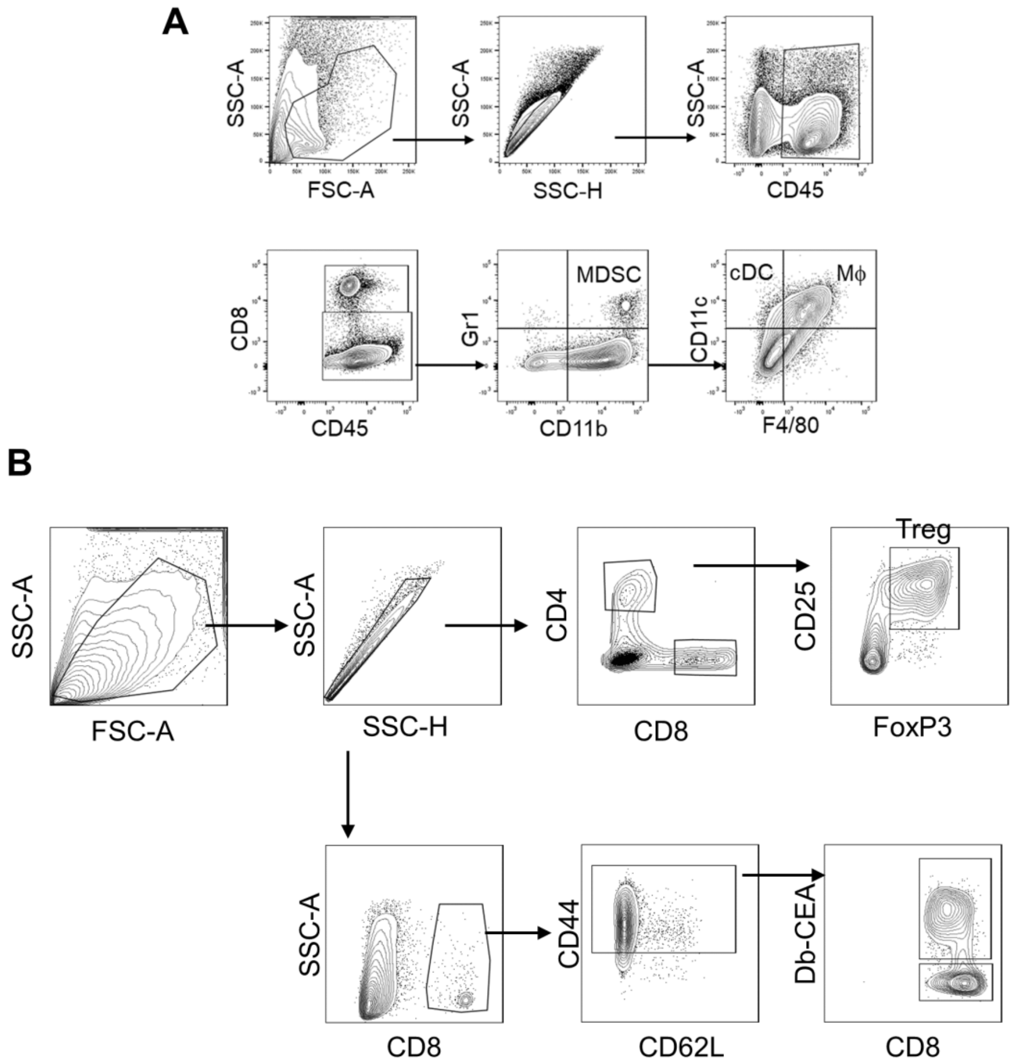

Figure S2. **Flow cytometric analysis of myeloid populations, Treg and T cells in MC38-CEA2 tumor. (A)** Representative FACS plots with gating strategy to identify MDSC (CD45+CD8-CD11b+Gr1+), Macrophages (CD45+CD8-CD11b+F4/80+CD11c+) and conventional DCs (CD45+CD8-CD11b+CD11c+F4/80-). **(B)** Representative FACS plots with gating strategy to identify CD4+ T cells, Treg (CD4+FoxP3+CD25+) and tumor specific CD8+ T cells (CD8+CD44+Db-CEAtet+).

Figure S3

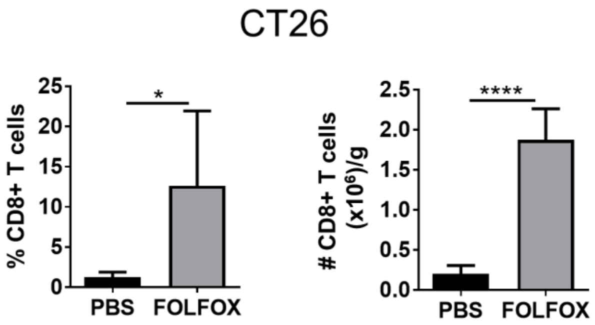

Figure S3. **FOLFOX treatment is associated with increased tumor infiltrated CD8 T lymphocytes in CT26 tumor model.** Examination of tumor-infiltrating CD8 T cells harvested 4 days after the third treatment in experiments performed as in Figure 1 by flow cytometry. CT26 tumor were collected and analyzed for frequency and number of CD8 T cells per gram of tumor tissue. Representative data of two independent experiments with each group containing 4-6 mice. *$p<0.05$, ****$p<0.0001$.

Figure S4

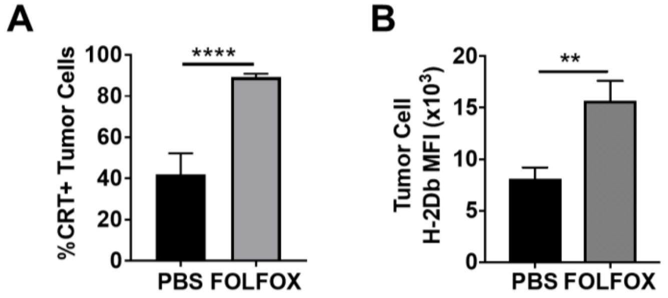

Figure S4. FOLFOX treatment induces immune cell death and augments MHC expression on tumor cells. MC38-CEA2-GFP cells were implanted subcutaneously in naïve C57BL/6 mice. After tumor was established, mice were treated with FOLFOX or PBS for three rounds. Tumor cells from PBS or FOLFOX treated animals harvested 4 days after the third treatment were analyzed for calreticulin (CRT) and MHC class I (MHCI, Db) expression on live tumor cells. Viable tumor cells were determined first by using a forward and side scatter live gate and second, by gating on GFP+ CD45- tumor cells. **(A)** Frequency of CRT expression and **(B)** H-2Db expression level by tumor cells. Representative data of n≥3 experiments with each group containing 4-6 mice. **p<0.01, ***p<0.001.

Figure S5

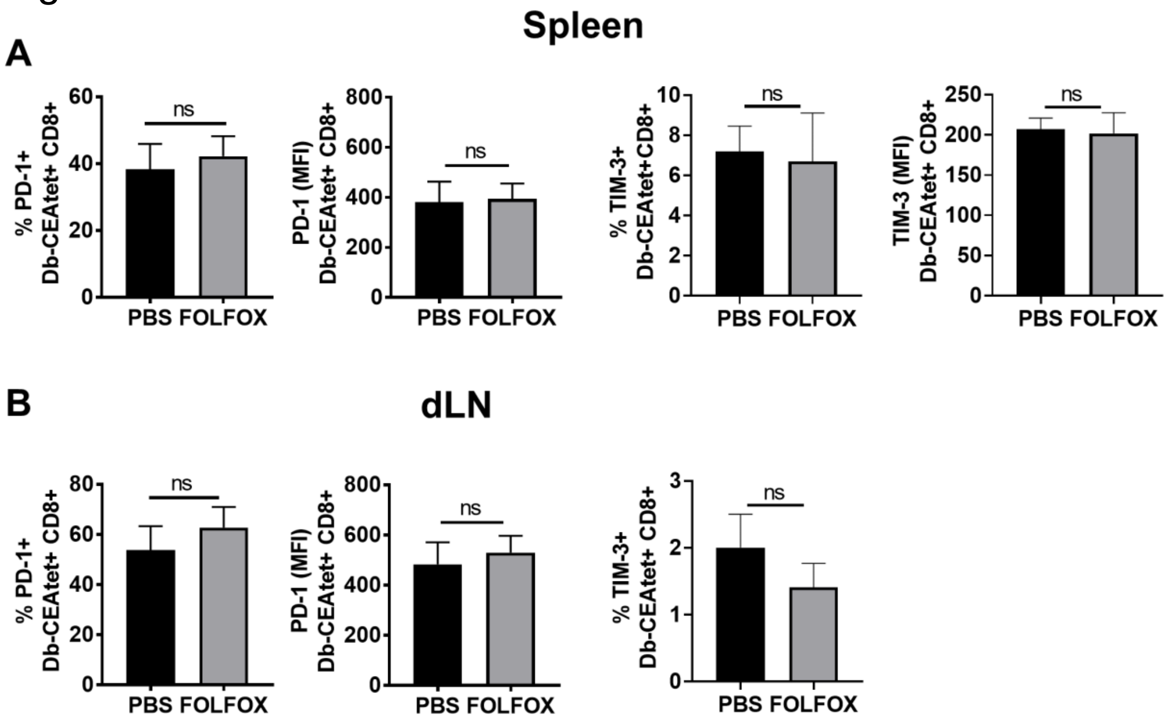

Figure S5. **Spleen and tumor dLN CD8 T lymphocytes express similar level of PD-1 and TIM-3 between PBS and FOLFOX treatment.** Spleen and dLN from PBS or FOLFOX treated animals harvested 4 days after the third treatment were analyzed for indicated phenotype. **(A)** Spleen CD8 T cells and (**B**) dLN CD8 T cells were gated on CD44hiDb-CEAtet+ population for the expression of PD-1 and TIM-3 in frequency and MFI. The TIM-3 MFI in dLN was below the detection.

Figure S6

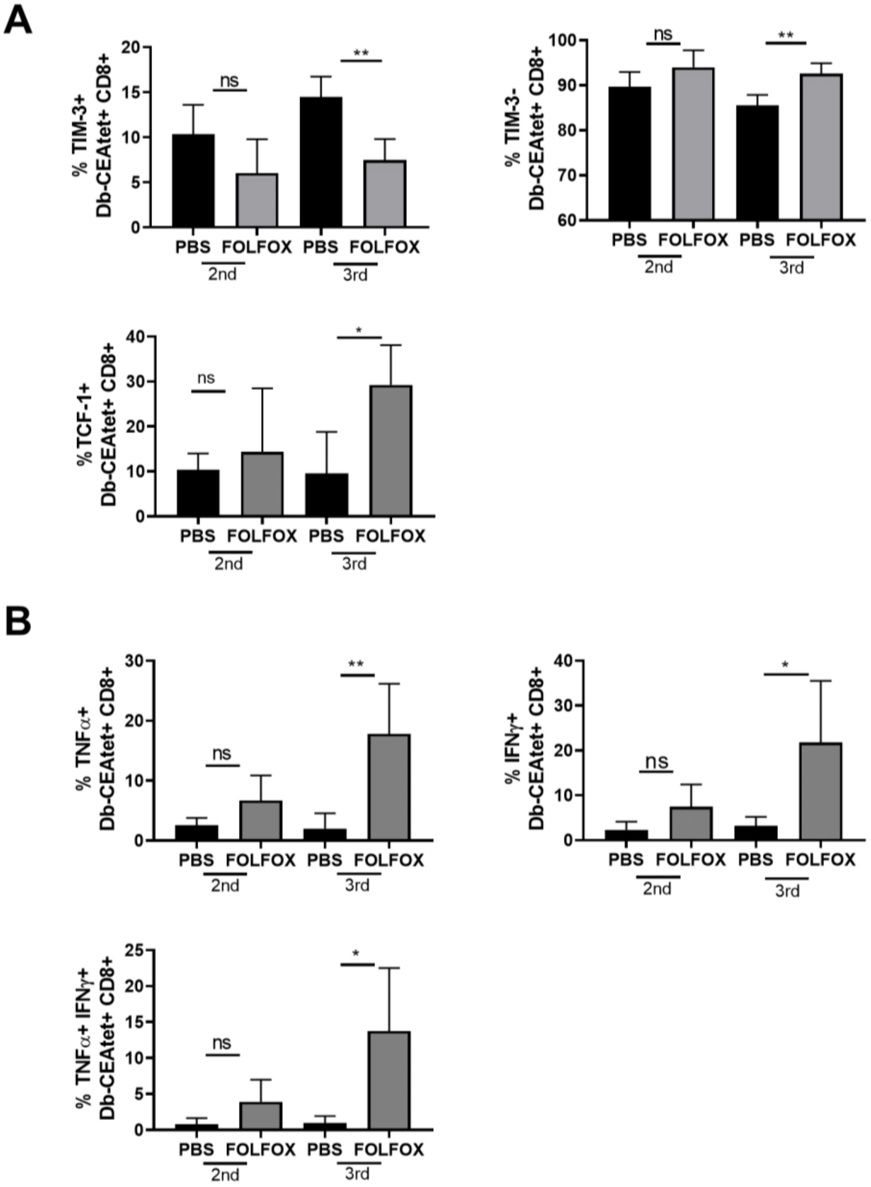

Figure S6. **The frequency of less exhausted tumor specific CD8 T cells increase between the second and third treatment of FOLFOX.** Tumor antigen specific CD8 T cells from PBS or FOLFOX treated animals harvested after the second and the third treatment were analyzed for indicated phenotype. Tumor antigen specific CD8 T lymphocytes were gated on CD8+CD44hiDb-CEAtet+ population. **(A)** Graphs show frequency of tumor antigen specific CD8 T cells that are positive for TIM-3, or negative for TIM-3, or positive for TCF-1. **(B)** Tumor cells were re-stimulated with PMA and ionomycin for 5 hour in the presence of Golgiplug for *ex vivo* T cell function analysis. Graphs show frequency of CEA antigen specific CD8 T cells that are positive for the expression of the cytokine indicated. Representative data of ≥2 with each group containing 4-6 mice. *p<0.05, **p<0.01.

Figure S7

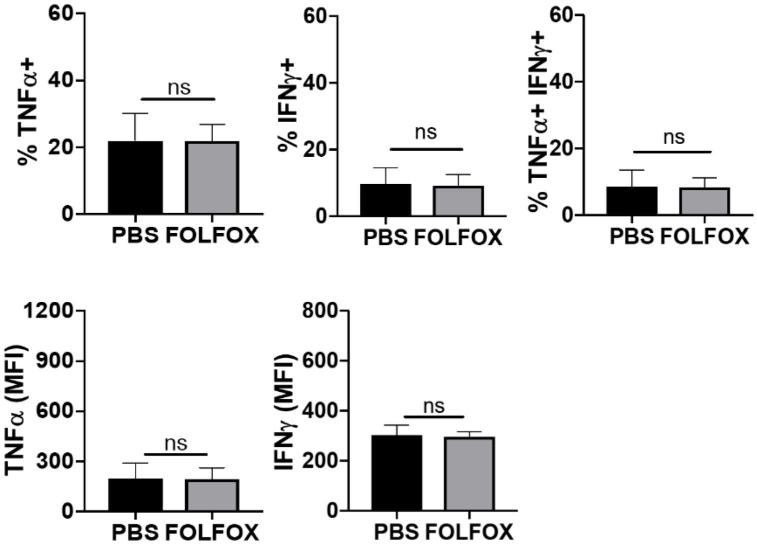

Figure S7. Spleen CD8 T lymphocytes in FOLFOX treated mice produce similar level of cytokines upon stimulation compared to control mice. Flow cytometric analysis of spleen harvested 4 days after the third treatment in experiments performed as in Figure 1. Spleen cells were stimulated with PMA and ionomycin for 5 hour in presence of Golgiplug for *ex vivo* T cell function analysis. Tumor antigen specific CD8 T cells were identified by CD8+ CD44hi Db-CEAtet+ cells in spleen samples. Graphs show frequency of tumor antigen specific that are positive for the expression of the cytokine indicated and levels of cytokines (MFI) expressed by tumor antigen specific.
